# Visible Portion of Anterior Teeth at Rest and Analysis of Different Smile Characteristics in the Saudi Population of the Jeddah Region

**DOI:** 10.1155/2020/8859376

**Published:** 2020-11-26

**Authors:** Mohamed Abdelmageed Awad, Doaa Saleh Alghamdi, Aljawharah Talal Alghamdi

**Affiliations:** ^1^Oral and Maxillofacial Prosthodontics Department, Faculty of Dentistry, King Abdulaziz University, Jeddah, Saudi Arabia; ^2^Fixed Prosthodontic Department, Faculty of Dentistry, Tanta University, Tanta, Egypt; ^3^Faculty of Dentistry, King Abdulaziz University, Jeddah, Saudi Arabia

## Abstract

This cross-sectional study aimed to determine the degree of visibility of maxillary and mandibular anterior teeth at rest in different age groups and to evaluate the smile line, smile arc, and number of maxillary teeth displayed during smiling among the Saudi population visiting King Abdulaziz University Dental Hospital, Jeddah, Saudi Arabia. A total of 157 participants (77 males and 80 females) were included in this study with an age range of 19 to 69 years. All participants had maxillary and mandibular anterior teeth without restorations. Crown length and visible portions of anterior teeth at rest were measured using a Fowler Electronic Digital Caliper. Three measurements of each variable were recorded by two independent dental interns and calibrating their measurements was performed. Smile line, smile arc, and number of teeth displayed during smiling were examined. The collected data were statistically analyzed using SPSS software. It was found that the average clinical crown lengths in the maxillary lateral incisor, mandibular central incisor, and canine were significantly higher in males than females. At rest position, females displayed more maxillary central and lateral incisors. The displayed length of maxillary teeth at rest is inversely proportional to increasing age; the reverse occurs in mandibular teeth. The common visibility of maxillary teeth during smiling was from the second bicuspid to its counterpart. Average smile line and consonant smile arc were the most common characteristics. It was concluded that both age and gender affect the characteristics of tooth display at rest and in smiling. These effects should be considered during treatment planning and restoration of anterior teeth to obtain a more predictable esthetic outcome.

## 1. Introduction

The lips act as a frame for the smile. The length and curvature of the lips significantly influence tooth display at rest and during functioning. The extent of incisal tooth display at rest is an important esthetic factor in evaluating the outcome of fixed and removable prosthodontic procedures [1]. Accepted prosthodontics guidelines recommend displaying 2–4 mm in the arrangement of upper incisor teeth below the relaxed lip [2].

The smile plays a major role in the overall perception of physical attractiveness. Dentofacial esthetics and an attractive smile motivate patients to seek dental and prosthetic treatment to enhance their confidence, their career, and a more satisfying life [3]. Smile analysis helps in diagnosing and planning treatment, as it gathers information about the relationship between teeth and their surrounding soft tissue [4–6].

When the lips are functioning, two types of smiling can occur: social and spontaneous smiles. The social smile is voluntary and continuously repetitive but not related to emotions. Spontaneous smiles develop involuntarily and depend on emotions [7].

Awareness of various smile parameters, such as smile line and smile arc, is required to create an esthetic smile. Also, understanding the variables that may have an effect on the smile, such as age and gender, is quite important to reduce their effect on the final appearance [8]. The smile line is a curve passing from the tip of one canine to the tip of the other through the maxillary incisal margin of the anterior teeth [9]. The smile line is low if half the maxillary anterior teeth show in smiling and average when 1-2 mm of gingiva is displayed with the teeth, while it is considered high if a large part of the gingiva is displayed with the teeth [10]. On the other hand, the smile arc is the relationship between the incisal edge of the maxillary anterior teeth and the superior border of the lower lip [11]. A parallel relationship is more esthetic, known as a consonant smile arc [10, 11]. A nonconsonant smile arc is characterized by the maxillary incisal curvature being flatter than that of the lower lip or reversed; this is called a flat or reversed smile arc. The smile arc tends to be flat or reversed with worn dentition, a perception associated with old age [12].

Previous studies have reported that female subjects display significantly more upper gingival tissue during smiling than males, and this “gummy smile” is considered a female characteristic [13–15]. Owens et al. evaluated gingival display in the social smile of six racial groups—African American, Caucasian, Chinese, Hispanic, Japanese, and Korean—and found that African Americans displayed significantly more gingival tissue than other groups. They also reported that the amount of display of maxillary teeth is inversely proportional to increasing age and is accompanied concurrently with a gradual increase in the display of mandibular teeth [15]. Racial differences in the amount of maxillary central incisors displayed were also reported, with white Americans displaying more tooth surface than blacks [16, 17]. Linag et al. compared Chinese and Caucasian subjects and found that race and gender had significant effects on upper lip curvature and smile classifications [18]. In the Saudi population, one study revealed that the average smile line was more prevalent than other types [19]. Another study performed on the same population revealed that most participants had an average lip position and consonant smile arc. A limitation of this study was the small sample size (30 participants) [20].

Thus, the purpose of this study was to assess the amount of display of maxillary and mandibular anterior teeth in the at rest position and to evaluate smile line and smile arc in the Saudi population to act as a guide for esthetic restoration of anterior teeth. The null hypothesis was that age and gender have no effect on the esthetic components.

## 2. Materials and Methods

The participants in our study were selected from the Saudi population attending King Abdulaziz University Dental Hospital (KAUDH) in Jeddah between January and March of 2020, aged 19 years and above. A total of 157 participants (77 males and 80 females) were included using the convenience sampling technique. Ethical approval (No. 161-11-19) was obtained from the Ethics Committee of the Faculty of Dentistry, KAU, and written informed consent was obtained from participants. All participants had maxillary and mandibular natural anterior teeth with no caries, restorations, extreme occlusal wear, extrusion, obvious deformities, periodontal disease, or tooth mobility. The exclusion criteria were facial asymmetry, orthodontic treatment, deep or open bite, increased overjet, anterior crossbite, history of congenital anomalies, lip trauma, and orthognathic or periodontal surgery in the anterior region. Participants were divided into five groups by age as follows:

  Group 1: range between 19 and less than 25 years
  Group 2: range between 25 and less than 35 years
  Group 3: range between 35 and less than 45 years
  Group 4: range between 45 and less than 60 years
  Group 5: 60 years and above

**Figure 1:**
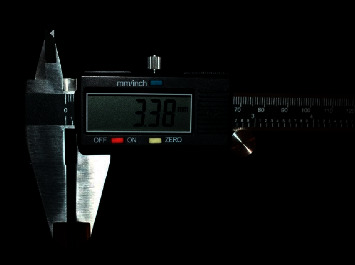
Fowler Electronic Digital Caliper used in this study.

Participants signed a consent form before examination and data collection. Measurements were made using a digital caliper (Fowler Electronic Digital Caliper, Kevelaer, Germany) to the nearest tenth of a millimeter. The measuring gauge had a resolution of 0.01 mm, and measured dimensions were recorded to this degree of accuracy (Figure 1). Examination and data collection were performed by two independent dental interns, and measurements for specific lengths were recorded three times.

Measurements were recorded in accordance with Al-Habahbeh et al. [21]. The visible portions of maxillary anterior teeth at rest were measured using internal edges of the caliper held vertically from the lower border of the upper lip to their incisal edges or cusp tips at the midpoint of each tooth. For mandibular teeth, measurements were taken from the upper border of the lower lip to their incisal edges or cusp tips, at the midpoint of each tooth at rest position. The clinical crown length of maxillary or mandibular anterior teeth was measured from its incisal edge or cusp tip to the highest or lowest point of the gingival margin, respectively. The measurement was recorded as zero if the tooth could not be seen, regardless of how short it was.

To begin, participants were instructed to sit in an upright position on an office chair with their feet on the ground, head positioned without support, and looking straight ahead. Examination of the smile line, smile arc, and the number of maxillary teeth displayed during social (forced) smile was conducted. The smile line was classified as low, average, high, or gummy. Smile arc was recorded as consonant, flat, or reversed. The number of maxillary teeth displayed during smiling was classified as follows: A, revealing up to the canines on both sides (3-3); B, revealing up to first premolars (4-4); C, revealing up to second premolars (5-5); D, revealing up to first molars (6-6); and E, revealing up to the second molars on both sides (7-7).

Data were collected and analyzed using SPSS version 21 (IBM Corp., Armonk, NY, USA). Chi-square, *t*-test, and ANOVA were used for statistical analysis, and a probability value of less than 0.05 was established as statistically significant.

## 3. Results

Interreliability between examiners was verified using paired student's *t*-test and found no significant differences between measurements (*p*=0.253), with a strong Cronbach's coefficient (0.997).

Age range in years and gender distributions of the participants are shown in Table 1 as numbers and percentages for the males, females, and total.  Table 2 shows the mean ± standard deviation (SD) of the clinical crown length (CCL) and vertical tooth display at rest (TDR) of maxillary and mandibular anterior teeth. Differences between male and female participants are also shown. The average CCLs of maxillary lateral incisor, mandibular central incisor, and canine were significantly higher in males than females. At rest position, females displayed more maxillary central and lateral incisors vertically than males, while males showed more maxillary canines than females. On the other hand, there was no significant difference between genders in the display of mandibular anterior teeth, although males showed more mandibular central and canine teeth than females (Table 2).

Table 3 shows the mean ± SD of CCL and TDR of maxillary and mandibular anterior teeth distributed among the five age groups and the differences between them. The maxillary TDR is inversely proportional to increasing age, whereas the mandibular TDR is directly proportional to it. Younger participants tend to show more maxillary anterior teeth, while older participants tend to show more mandibular anterior teeth. The average CCL of all anterior teeth was not affected by increasing age except in mandibular canines.

Table 4 shows the percentage of TDR in relation to CCL for male and female participants. Females displayed more labial surfaces of all anterior teeth at rest except maxillary canines and the mandibular central incisors and canines.

Table 5 shows the frequency of smile line, smile arc, number of teeth displayed during smiling, and gender-based differences in the various parameters. Gender was significantly associated with smile line and number of maxillary teeth displayed during smiling. More than half the participants (99, 63.1%) had an average smile line in nearly equal numbers of males and females. The same occurred for consonant smile arcs (105 participants, 66.9%). For maxillary teeth displayed during smiling, the C-smile was the most prominent in both genders, followed by the D-smile, while the A-smile was the least prominent in all participants.

## 4. Discussion

In this study, gender played a significant role in the TDR of maxillary central incisors. This agrees with previous findings by Connor and Moshiri [16] and Vig and Brundo [17]. Although both Al Wazzan [1] and Al-Habahbeh et al. [21] found that females exposed more maxillary central incisors than males, the differences were not significant. Variations among the measurements of TDR of maxillary central incisors have been reported previously. In our study, the mean ± SD of TDR for females' maxillary central incisors was 2.40 ± 0.79 mm, and in males it was 2.09 ± 0.92 mm (Table 2). Measurements for females and males in Connor and Moshiri's study were 4.09 ± 2.27 mm and 1.82 ± 2.80 mm, respectively [16], while measurements reported by Vig and Brundo were 3.40 mm in females and 1.91 mm in males [17]. On the other hand, measurements reported by Al-Habahbeh et al. were 3.02 ± 1.96 mm and 2.63 ± 1.15 mm, respectively [21]. Our study showed that males displayed significantly more maxillary canines than females, while prominent maxillary lateral incisors were associated with females. Moreover, there were no gender differences in visibility of the mandibular anterior teeth at rest, contrary to previous studies [1, 17, 21], which found that males showed significantly more mandibular anterior teeth than females. The disparity in measurements may be due to differences in the race of the population of each study, measurement techniques, lip length, and lip type. Previous measurement techniques were done either directly on the participants by ruler [17] and Bowley gauge [16] or indirectly using a digital video camera [18]. Our measurements were recorded using a digital caliper with a resolution of 0.01 mm, and each measurement was repeated three times.

Variations in tooth display have also been reported among subjects of different ages [17, 22–24]. Our results indicate that the display of maxillary central incisors at rest generally declines with increasing age, from 2.93 mm in Group 1 to zero at ages above 60 years (Group 5), while mandibular incisor display increases with advancing age from 0.89 mm (Group 1) to 2.14 mm in Group 5 (Table 3). Desai et al. reported a significant decrease in vertical display of maxillary incisors after the age of 40 [25]. This was attributed to loss of tonicity of the facial muscles and reduced elasticity of the upper lip [26]. With increasing age, sagging at the corners of the mouth was reported as a result of an increase in resting muscle lengths (levator anguli oris, zygomaticus minor, and zygomaticus major) [27].

One factor that should be considered during measurements is the percentage of TDR of maxillary anterior teeth in relation to CCL. We found that this percentage for maxillary central and lateral incisors in females was 23% and 19.9%, respectively, which is more than that in males (20% and 16.2%) (Table 4). Al-Habahbeh et al. [21], who investigated maxillary and mandibular anterior teeth display in the Jordanian population, found that these percentages for maxillary central and lateral incisors were 28.8% and 16% in females and 24.5% and 20% in males, respectively. This difference may be due to racial factors.

Examining 168 smiling photographs selected from Time magazine, Orce-Romero et al. considered gender to be one of the factors that affect the characteristics of smile [28]. This agreed with our study in which smile line and maxillary teeth displayed during smiling were significantly affected by gender. Our study evaluated the location of the smile line during social smiling in relation to the maxillary teeth (Table 5). More than half the participants (63.1%) showed an average smile line, while much fewer showed high and low smile lines (14.6% for both) and least was a gummy smile line (7.6%), predominantly in females (Table 5). This result is comparable with a previously reported study on the Turkish population ranging between 18 and 25 years of age in which the percentages of smile lines were 45.3% for average, 36.3% for low, and 18.4% for high smile lines [29]; they did not differentiate between high and gummy smile lines. This difference between results may be due to racial factors. Our study confirmed previous study [30], in which the gummy smile was predominantly a female characteristic. Our results were also comparable to another study conducted on the Saudi population ranging between 18 and 35 years of age in which the frequency of smile lines was 57.5% for average, 24.1% for high, and 18.4% for low smile lines [19]. However, most of their participants were males (95.2%), and they did not differentiate between high and gummy smile lines. These variations suggest the need to standardize parameters for comparison.

Parallelism of the upper incisal curve with an inner curvature of the lower lip was also assessed (Table 5). The literature suggests that the convex smile arc is more esthetic than the concave (reversed) arc [31]. In our results, the consonant (convex) smile arc was more prevalent in all participants evaluated (66.9%), whereas the reverse smile arc was least frequent (6.4%). This finding agrees with many studies [11, 25, 30, 32, 33]. Contrary to our results, Maulik and Nanda revealed that the straight smile arc was the most common finding observed in 49% of their participants, followed by consonant (40%) and reverse smile arc (10%) [4]. Some previous studies showed statistically significant differences between male and female participants regarding smile arc [11, 33]. However, no such difference was observed in our study.

Regarding the number of maxillary teeth displayed during smiling, we found that the C-smile was most common (42%), followed by the D-smile (35%). This result is similar to those of Maulik and Nanda [4] and Al-Johany et al. [32]. However, Khan et al. [11] found that the highest number of maxillary teeth displayed during smiling was from the first bicuspid to its counterpart (the B-smile). Our study suggests that males have wider smiles exposing more maxillary teeth than females in the social smile. This is in line with Khan et al. [11]. On the other hand, Nold et al. [33] reported no gender difference for the extent of teeth visible during smiling.

Several limitations were encountered in this study, including the limited number of participants due to the COVID-19 pandemic, which has had a major impact on healthcare workers, including dentists, who are exposed to great risk of COVID-19 infection due to direct exposure to patients' body fluids [34]. A recent review shows that COVID-19 has affected treatment protocols and attendance at dental clinics [35]. The second limitation is the lack of external validity, as it was conducted in Jeddah city only. Further studies are needed to assess smile line, smile arc, and number of maxillary teeth displayed during smiling in the Saudi population to render these results more generalizable.

## 5. Conclusion

The vertical display of anterior teeth at rest is influenced by age and gender. In addition, gender plays a role in the smile line level and the number of maxillary teeth displayed during smiling. Gummy smiles are more prevalent in females. The most common display of maxillary teeth during smiling was from the second bicuspid to its counterpart (C-smile), followed by the D-smile. These factors should be taken into consideration when planning esthetic restorations.

## Figures and Tables

**Table 1:** Age range in years and gender distribution of the participants.

	Male		Female		Total	
Age range (years)	Number	Percentage	Number	Percentage	Number	Percentage
19–<25	11	14	30	38	41	26
25–<35	30	39	15	19	45	29
35–<45	12	16	16	20	28	18
45–<60	19	25	17	21	36	23
≥60	5	6	2	3	7	4
Total	77	100	80	100	157	100

**Table 2:** Mean ± standard deviation (mm) of clinical crown length (CCL), vertical tooth display at rest (TDR), and the difference between male and female participants.

	Tooth		Male	Female	*P* value
Maxillary anterior teeth	Central incisor	CCL	10.49 ± 0.42	10.41 ± 0.57	0.29
		TDR	2.09 ± 0.92	2.40 ± 0.79	0.028^*∗*^
	Lateral incisor	CCL	9.16 ± 0.54	8.94 ± 0.57	0.01^*∗*^
		TDR	1.47 ± 0.68	1.78 ± 0.78	0.009^*∗*^
	Canine	CCL	10.36 ± 0.64	10.19 ± 0.64	0.09
		TDR	0.52 ± 0.44	0.36 ± 0.33	0.012^*∗*^

Mandibular anterior teeth	Central incisor	CCL	9.28 ± 0.47	9.04 ± 0.43	0.001^*∗*^
		TDR	1.47 ± 0.72	1.34 ± 0.70	0.254
	Lateral incisor	CCL	9.27 ± 0.43	9.21 ± 0.39	0.38
		TDR	1.17 ± 0.61	1.24 ± 0.65	0.698
	Canine	CCL	10.98 ± 0.55	10.79 ± 0.46	0.02^*∗*^
		TDR	0.71 ± 0.59	0.67 ± 0.53	0.48

^*∗*^ Significant <0.05.

**Table 3:** Mean ± standard deviation (mm) of clinical crown length (CCL) and vertical tooth display at rest (TDR) distributed on the five age groups and difference between them using ANOVA.

	Tooth		Age groups (years)					
			19–<25	25–<35	35–<45	45–<60	Above 60	*P* value
Maxillary anterior teeth	Central incisor	CCL	10.57 ± 0.38	10.46 ± 0.54	10.4 ± 0.55	10.39 ± 0.49	10.24 ± 0.7	0.36
		TDR	2.93 ± 0.44	2.57 ± 0.49	2.32 ± 0.53	1.47 ± 0.54	0	0.001^*∗*^
	Lateral incisor	CCL	9.05 ± 0.52	9.0 ± 0.56	9.19 ± 0.61	8.95 ± 0.58	9.21 ± 0.65	0.46
		TDR	2.19 ± 0.75	1.73 ± 0.53	1.65 ± 0.52	1.17 ± 0.40	0	0.001^*∗*^
	Canine	CCL	10.31 ± 0.46	10.19 ± 0.56	10.53 ± 0.99	10.21 ± 0.52	9.86 ± 0.53	0.07
		TDR	0.43 ± 0.38	0.55 ± 0.39	0.49 ± 0.33	0.35 ± 0.43	0	0.004^*∗*^

Mandibular anterior teeth	Central incisor	CCL	9.13 ± 0.49	9.15 ± 0.46	9.3 ± 0.53	9.04 ± 0.35	9.29 ± 0.48	0.24
		TDR	0.89 ± 0.41	1.12 ± 0.50	1.54 ± 0.60	2.09 ± 0.65	2.14 ± 0.28	0.001^*∗*^
	Lateral incisor	CCL	9.34 ± 0.43	9.11 ± 0.38	9.27 ± 0.43	9.24 ± 0.36	9.43 ± 0.52	0.08
		TDR	0.89 ± 0.42	0.89 ± 0.45	1.32 ± 0.64	1.68 ± 0.55	2.13 ± 0.33	0.001^*∗*^
	Canine	CCL	10.83 ± 0.43	10.95 ± 0.5	10.95 ± 0.58	10.89 ± 0.47	10.29 ± 0.71	0.02^*∗*^
		TDR	0.41 ± 0.31	0.54 ± 0.39	0.72 ± 0.50	0.90 ± 0.40	2.04 ± 1.05	0.001^*∗*^

^*∗*^ Significant <0.05.

**Table 4:** Percentage of TDR in relation to its CCL for male and female participants.

	Maxillary anterior teeth			Mandibular anterior teeth		
	Central incisor	Lateral incisor	Canine	Central incisor	Lateral incisor	Canine
Female	23.0	19.9	3.5	14.8	13.5	6.3
Male	20.0	16.2	5.0	16.0	12.6	6.5

**Table 5:** Frequency of smile line, smile arc, maxillary teeth displayed during smiling of total subjects (males and females), and gender-based differences for various parameters using Pearson chi-square test.

		Total subjects		Male			Female			*P* value
		*N*	% of total	*N*	% of total	% within gender	*N*	% of total	% within gender	
Smile line level	Average	99	63.1	50	31.8	64.9	49	31.2	61.3	0.032^*∗*^
	Low	23	14.6	17	10.8	22.1	6	3.8	7.5	
	High	23	14.6	6	3.8	7.8	17	10.8	21.3	
	Gummy	12	7.6	4	2.5	5.2	8	5.1	10	

Smile arc	Consonant	105	66.9	52	33.1	67.5	53	33.8	66.3	0.140
	Flat	42	26.8	23	14.6	29.9	19	12.1	23.8	
	Reversed	10	6.4	2	1.3	2.6	8	5.1	10	

Maxillary teeth displayed during smiling	A-smile	2	1.3	0	0	0	2	1.3	2.5	0.002^*∗*^
	B-smile	25	15.9	5	3.2	6.5	20	12.7	25	
	C-smile	66	42	37	23.6	48.1	29	18.5	36.3	
	D-smile	55	35	33	21	42.9	22	14	27.5	
	E-smile	9	5.7	2	1.3	2.6	7	4.5	8.8	

^*∗*^ Significant <0.05.

## Data Availability

The data used to support this study are available upon request.
